# Observations on Country Practice1Read before the Fifteenth Annual Meeting of the Iowa State Veterinary Medical Association, Cedar Rapids, Iowa, January 14 and 15, 1903.

**Published:** 1903-03

**Authors:** S. H. Bauman

**Affiliations:** Birmingham, Iowa


### OBSERVATIONS ON COUNTRY PRACTICE.[1]

By S. H. Bauman, D.V.S.,
BIRMINGHAM, IOWA.

Having had experience in both city and country practice, it occurred to me that I might be able to give a few items that might be of interest at least to those who have had no experience in country practice, and perhaps be able to point out the large, pleasant, and lucrative field still open to our profession. A little over five years ago, when I left the city, I hardly expected to follow the profession except as a side-line, having decided to make farming and stock-raising my future business. I was of the opinion that in a farming community there would be little or no sickness among stock, owing to the natural surroundings, such as exercise, forage, good care, and experience gained by farmers in handling stock from their youth up, and hardly expected to find anything outside of the simple cases and occasional cases of obstetrics; but, to my surprise, I found the field a large one, with perhaps a greater variety of cases than are found in a city practice. In regard to the side line, inside of a year I had to turn over the management of the farm to other members of the family, my practice wholly occupying my time. I have found the field both pleasant and profitable, and find the farmers take a great deal of interest in the treatment and operations, and, as a class, are exceedingly well posted on disease and treatment in general, much more so than their city cousins. This knowledge results from reading the best farm and stock papers, and reports from the experiment stations and Department of Agriculture. To illustrate: When first I used the Schmidt method in the treatment of parturient apoplexy for quite an old farmer he watched me with interest for some time, then said: "That's new in the United States. I don't see how they got it over here so soon. Why, it's only a short time since Dr. Schmidt was making his experiments in Europe." Asking him how he came to know so much about it, he said he had got it from the Government reports and papers.

The field is a nice one, in that you get good treatment from your patrons. Talk about the cooks and good meals! they make you feel that the best is none too good for the "horse-doctor." It is not the dollar you earn but the dollar you save that counts in the end, and that is what I like in a country practice. Your table is

[1] Read before the Fifteenth Annual Meeting of the Iowa State Veterinary Medical Association, Cedar Rapids, Iowa, January 14 and 15, 1903.

almost wholly supplied from your own garden and farm. The feed for the horses is not a direct outlay, as it is all produced on the farm, and this is quite an item when you run an infirmary. Probably as great a source of profit as any single item is being able to pick up bargains in young stock slightly blemished, requiring time and not much treatment, allowing it to run on the farm and develop. In a country practice a man should be fairly well posted in diseases and treatment of all domesticated animals, as many of your calls are to cattle and sheep. Creameries and the two crops of sheep—*i. e.*, wool and lambs—make money rather plenty in the country, making collections fairly easy and prompt. Conditions have vastly changed from what they were under the old system of farm life, when the whole crop was held and marketed but once a year. In the richer and better farming communities the stores run delivery-wagons, delivering groceries to the farm-home regularly and collecting the farm produce. New houses are being erected everywhere, with furnaces, baths, acetylene lights, and all modern improvements. The tendency is for better schools, and all the towns and villages have a good system of graded schools. It is really hard to find a house that is more than a mile from a rural telephone line, which keeps you in close touch with your patrons and all the towns and villages in the country in which you live. This, with rural mail delivery, makes the country one of the most desirable places to reside. The tendency in all farming communities is for better roads, and already, when the material can be procured cheaper, all bad pieces of roadway are being macadamized. We also have many places in our State where interurban electric lines are being built, while many other lines are being projected, and the farmers are ready to assist in the building of these by granting right of way and voting tax to help pay for their construction. Farmers, as a class, are comfortably fixed, while many are quite wealthy, and, as a rule, are contented. The society is of the best. The young people have their parties, socials, singing-schools, and literary societies at nearly every school-house, while in the towns, which are only five or six miles apart, they have lecture courses, light operas, etc. The older portions of the community have their birthday dinners and other social meetings without number, and all who wish to attend are made welcome and cordially treated.

The best part of a country practice with a home on a farm is the healthy and moral surroundings of your family, who are inspired by the natural environments to become industrious, rugged citizens, all of them employed pleasantly, and, as a rule, contented. The day of the "clod-hopper" and "old hayseed" is past. The farmers' sons and daughters are, as a rule, graduates of the graded schools, and many of the most successful business and professional men have developed their fine physical constitutions and breadth of brain on the farm. The farmers are waiting anxiously for men of our profession to locate among them, and if we are worthy of their confidence and qualified they will do all in their power to hold us there by giving us their patronage and encouragement. I do not wish you for a moment to think everything is flowing with milk and honey in the country. In certain times of the year roads are very bad. We sometimes have partial failure of crops and many other discouraging things, but, on the whole, I consider it pleasant, profitable, and a wide field to develop.

